# Supplementary material for: Mechanisms governing the pioneering and redistribution capabilities of the non-classical pioneer PU.1
Source: Nat Commun. 2020 Jan 21;11:402. doi: 10.1038/s41467-019-13960-2 (PMC6972792; doi:10.1038/s41467-019-13960-2)
Supplement: Supplementary file 7 — Source data [file 41467_2019_13960_MOESM7_ESM.zip › Source_Data/FigureS2/FigureS2B_MotifScanOutput/KmeansCluster3/homerResults/motif1.similar.html]

motif1

## Information for motif1

C
G
T
A
C
G
T
A
C
G
T
A
T
C
A
G
C
T
G
A
C
T
A
G
A
C
T
G
C
G
T
A
C
G
T
A
T
A
C
G
A
C
G
T
T
A
C
G
  
Reverse Opposite:  

A
T
G
C
T
G
C
A
A
T
G
C
A
C
G
T
A
C
G
T
A
G
T
C
A
G
T
C
G
A
C
T
A
G
T
C
G
C
A
T
G
C
A
T
G
C
A
T
  

|  |  |
| --- | --- |
| p-value: | 1e-2802 |
| log p-value: | -6.453e+03 |
| Information Content per bp: | 1.676 |
| Number of Target Sequences with motif | 4156.0 |
| Percentage of Target Sequences with motif | 87.15% |
| Number of Background Sequences with motif | 4284.1 |
| Percentage of Background Sequences with motif | 10.10% |
| Average Position of motif in Targets | 101.8 +/- 26.0bp |
| Average Position of motif in Background | 97.9 +/- 79.4bp |
| Strand Bias (log2 ratio + to - strand density) | 0.1 |
| Multiplicity (# of sites on avg that occur together) | 1.23 |
| Motif File: | file (matrix) reverse opposite |

### Similar de novo motifs found

|  |  |  |  |  |  |  |  |
| --- | --- | --- | --- | --- | --- | --- | --- |
| Rank | Match Score | Redundant Motif | P-value | log P-value | % of Targets | % of Background | Motif file |
| 1 | 0.980 | C T G A C G T A T C A G C T G A C T A G A C T G C G T A C G T A T A C G G C A T T A C G | 1e-2591 | -5968.328113 | 82.45% | 9.21% | motif file (matrix) |
| 2 | 0.986 | T C G A T C G A C T G A C T G A C T G A T C A G C T G A C T A G A C T G C G T A C G T A T A C G C G A T T A C G | 1e-2444 | -5628.107789 | 78.47% | 8.37% | motif file (matrix) |
| 3 | 0.981 | T C G A C T G A C T G A C G T A T C A G T C G A C T A G A T C G C G T A C G T A T A C G G C A T T A C G | 1e-2438 | -5614.759666 | 82.72% | 10.67% | motif file (matrix) |
| 4 | 0.964 | C G T A T C A G T C G A T C A G A C T G C G T A G T C A T A C G G C A T A T C G | 1e-2294 | -5283.478040 | 79.81% | 10.33% | motif file (matrix) |
| 5 | 0.932 | T C A G C T G A A C T G A C T G C G T A C G T A T A C G G C A T T A C G | 1e-2140 | -4929.630606 | 73.62% | 8.50% | motif file (matrix) |
| 6 | 0.915 | C T G A A C T G A C T G C G T A C G T A A T C G A C G T A T C G | 1e-1974 | -4545.762806 | 68.48% | 7.52% | motif file (matrix) |
| 7 | 0.936 | C T G A A T G C C G A T A C G T A G T C A G T C G A C T A G T C G C A T G C A T G C A T | 1e-1929 | -4442.086007 | 92.83% | 25.04% | motif file (matrix) |
| 8 | 0.933 | C T G A T C G A C G T A T C A G T C G A C T A G A C T G C G T A C G T A T A C G G A C T | 1e-1927 | -4437.208714 | 79.28% | 13.77% | motif file (matrix) |
| 9 | 0.944 | C T G A C G T A T C A G C T G A C T A G T A C G T G C A C G T A T A C G G C A T C T A G T C G A | 1e-1918 | -4418.351943 | 94.76% | 27.47% | motif file (matrix) |
| 10 | 0.920 | C G A T C A G T A T C G C T G A A C T G A C T G C G T A C G T A A T C G C G A T | 1e-1804 | -4155.113289 | 91.28% | 25.40% | motif file (matrix) |
| 11 | 0.908 | T C A G T C G A T C A G A T C G T C G A T G C A T A C G G A C T | 1e-1685 | -3880.658244 | 80.60% | 17.77% | motif file (matrix) |
| 12 | 0.906 | C T G A C T G A T A C G T C G A C T A G T C A G G T C A C T G A T A C G G C A T | 1e-1659 | -3821.334579 | 76.14% | 14.97% | motif file (matrix) |
| 13 | 0.879 | A C T G A C T G C G T A C G T A A C T G A C G T A T C G | 1e-1595 | -3673.319149 | 63.56% | 8.63% | motif file (matrix) |
| 14 | 0.849 | T C A G A C T G C G T A G T C A T A G C A C G T T C A G | 1e-1220 | -2811.375822 | 88.57% | 33.69% | motif file (matrix) |
| 15 | 0.874 | G A C T T C G A A G T C A C G T A C G T A G T C A G T C G A C T A G T C G C T A C A G T G A C T G A C T | 1e-1101 | -2537.390439 | 54.48% | 9.56% | motif file (matrix) |
| 16 | 0.870 | C T G A C T G A C G T A T C A G T C A G C T A G C T A G C G T A C G T A T A G C | 1e-1086 | -2502.068514 | 68.19% | 18.09% | motif file (matrix) |
| 17 | 0.863 | A T G C G A C T A C G T A G T C G A T C G A C T A T G C G A C T G A C T | 1e-1081 | -2491.273259 | 71.92% | 20.95% | motif file (matrix) |
| 18 | 0.888 | A T G C C T G A A T G C A G C T C G A T A T G C A G T C A C G T A G T C | 1e-992 | -2286.337786 | 63.20% | 16.28% | motif file (matrix) |
| 19 | 0.880 | C G T A G T C A C A G T T C A G C G T A C T A G T A C G T G C A C G T A A T C G C G A T C A G T C T G A | 1e-955 | -2199.488160 | 62.32% | 16.36% | motif file (matrix) |
| 20 | 0.782 | A G T C C T G A A G T C G C A T C A G T A T G C T A G C G T C A G A T C | 1e-948 | -2184.694632 | 68.38% | 20.81% | motif file (matrix) |
| 21 | 0.853 | A T G C A G C T A C G T A G T C A G T C A G C T A G T C | 1e-902 | -2077.652845 | 57.01% | 13.86% | motif file (matrix) |
| 22 | 0.854 | A T G C A C G T A C G T T A G C A G T C A G C T A T G C G C A T | 1e-852 | -1963.066678 | 75.91% | 29.35% | motif file (matrix) |
| 23 | 0.831 | A C G T A C G T A G T C A T G C A G C T A T G C G A C T A C G T | 1e-827 | -1904.847091 | 52.84% | 12.59% | motif file (matrix) |
| 24 | 0.809 | T C A G T C A G A C T G T C G A G T C A T A G C C A G T T A C G | 1e-572 | -1317.605381 | 43.82% | 11.79% | motif file (matrix) |
| 25 | 0.819 | G C A T C T G A A G T C G C A T A C G T A G T C A G T C A C G T A G T C A C G T A G C T A G C T G A C T G A C T | 1e-532 | -1226.694960 | 25.08% | 3.26% | motif file (matrix) |
| 26 | 0.829 | C A T G C G T A G T C A C G T A A C T G C T A G C T A G C T A G G T C A C T G A T C A G G C A T A T G C G C T A | 1e-488 | -1125.499995 | 28.39% | 5.12% | motif file (matrix) |
| 27 | 0.829 | C G A T T G C A T G A C C T G A A C T G A C T G C G T A G T C A A T C G C G T A T C G A T G C A T C A G | 1e-453 | -1043.505980 | 42.55% | 13.54% | motif file (matrix) |
| 28 | 0.759 | A C T G G A T C G A C T C G T A T A C G A T G C G T C A A T G C G A C T C A G T A G T C G A T C G T A C | 1e-295 | -680.220671 | 18.22% | 3.34% | motif file (matrix) |
| 29 | 0.781 | A G C T A G C T C A G T T C A G A G T C G C A T A C G T G A T C G A T C G A T C G T A C G A C T G A C T G A C T | 1e-253 | -583.044797 | 26.11% | 8.22% | motif file (matrix) |
| 30 | 0.846 | A C G T T A G C G C T A A T G C A C G T A C G T A G T C A G T C G A C T G A C T G T C A T G C A T C G A | 1e-246 | -567.726577 | 11.43% | 1.33% | motif file (matrix) |
| 31 | 0.760 | A C G T C T A G C T A G A C T G A C T G C G T A C G T A | 1e-227 | -523.904725 | 42.23% | 20.28% | motif file (matrix) |
| 32 | 0.775 | C G T A G T A C G T C A A C T G A C T G T G C A G T C A T C A G A G C T | 1e-224 | -516.089019 | 14.09% | 2.59% | motif file (matrix) |
| 33 | 0.824 | A G C T A G C T A G C T C G A T T C A G C T G A C T A G C T A G C G T A C G T A T A C G G C A T A T C G T G C A | 1e-196 | -451.441741 | 10.42% | 1.50% | motif file (matrix) |
| 34 | 0.754 | C T A G C T A G A C T G C T A G C G T A A C T G A C T G C G T A C T G A T C A G C G A T C T A G C A T G | 1e-152 | -351.550363 | 10.17% | 1.97% | motif file (matrix) |
| 35 | 0.776 | C T G A C T G A C T G A A G T C G T C A C T G A A C T G A C T G C G T A C G T A C T A G C G A T A T C G T C A G | 1e-32 | -75.711199 | 1.53% | 0.18% | motif file (matrix) |
